# Addressing the relationship between perceived fear of COVID-19 virus infection and emergence of burnout symptoms in a sample of Egyptian physicians during COVID-19 pandemic: a cross-sectional study

**DOI:** 10.1186/s43045-020-00079-0

**Published:** 2020-12-17

**Authors:** Mohamed Abdelghani, Hayam M. El-Gohary, Eman Fouad, Mervat S. Hassan

**Affiliations:** 1 Psychiatry Department, Faculty of Medicine, Zagazig University, PO Box 44519, Zagazig, Egypt; 2 2015-2016 Hubert H. Humphrey Fellowship, Department of Psychology, Virginia Commonwealth University, Richmond, VA USA

**Keywords:** Fear of COVID-19 virus infection, Burnout syndrome, Quality of life, Egypt, Physicians

## Abstract

**Background:**

Physicians during the COVID-19 pandemic are working under relentless stress. This study aimed to identify the impact of the perceived fears of COVID-19 virus infection on the quality of life and the emergence of burnout syndrome among physicians in Egypt during the COVID-19 outbreak. This cross-sectional study was conducted between May 10th and June 9th, 2020, and included 320 Egyptian physicians who were working during the outbreak of the COVID-19 pandemic. The participants were interviewed using the Fear of COVID-19 scale (FCV-19S), Hospital Anxiety and Depression Scale (HADS), Maslach Burnout Inventory, and World Health Organization Quality of Life Scale (WHOQOL-BREF) for assessment of the perceived fears of COVID-19 virus infection, associated anxiety and depressive symptoms, burnout symptoms, and quality of life, respectively.

**Results:**

Overall, most physicians were females (63%). Ideas about death, moderate-to-severe anxiety, and depressive symptoms were reported by 11, 28, and 29% of physicians, respectively. For burnout symptoms, high emotional exhaustion, high depersonalization, and low personal accomplishment were reported by 20, 71, and 39% of physicians, respectively. The perceived fear of COVID-19 virus infection was positively correlated with anxiety, depression, and burnout emotional exhaustion, and depersonalization symptoms, and negatively correlated with personal accomplishment and all quality of life domains.

**Conclusions:**

Egyptian physicians experienced higher levels of COVID-19-related fears, anxiety, and depressive and burnout symptoms. There was a robust correlation between these perceived fears, and higher burnout symptoms, and poor quality of life among physicians. Specific interventions should be tailored to minimize the physical and mental burdens on the physicians during the COVID-19 pandemic.

## Background

Coronavirus (COVID-19) outbreak is first recognized in Wuhan, China, and then spread worldwide. The virus is principally transmitted by respiratory droplets and close contact [1, 2]. Despite the shortage of medical staff specialized in the management of infectious diseases, health care providers, particularly physicians, are at the front lines exerting great and continuous efforts to face and manage the increased numbers of patients infected with COVID-19 virus infection [3, 4].

Studies have indicated high rates of symptoms of burnout syndrome (BOS) among working physicians. Almost one-third of physicians have reported BOS symptoms at certain points during their lives [5]. BOS has three interrelated dimensions namely emotional exhaustion, depersonalization, and personal achievement. Prolonged exposure to stress would usually result in emotional exhaustion and manifested as a lack of work enthusiasm, helplessness, feeling trapped, and overwhelmed. Physicians may develop a negative attitude toward their colleagues, indifferently treat their patients, or be withdrawn from their professional responsibilities leading to a state of depersonalization and lack of personal achievement [6].

Numerous studies confirmed the suffering of healthcare providers who experienced high levels of depression and anxiety during the COVID-19 outbreak [7, 8]. Moreover, physicians were exposed to high levels of distress at work in their usual life, and this persistent pressure might lead to emotional exhaustion, mental, and/or physical distress. On the career level, BOS might be associated with an increased incidence of medical errors and job dissatisfaction, and even early retirement [9, 10]. Physicians who could not tolerate workloads were more likely to report poor quality of life (QoL), particularly the physical and psychological domains of their lives [11].

To our knowledge, studies examining the psychological impact of COVID-19 pandemic among medical staff particularly physicians in Egypt are scarce, if any. In this study, our objectives are to assess the perceived fears of COVID-19 virus infection (FCV) and to evaluate its impact on the emergence of symptoms of BOS and the various QoL domains among physicians in Egypt during the COVID-19 outbreak.

## Methods

### Study setting

The physicians enrolled in this study were working during the outbreak of the COVID-19 pandemic in the largest three governmental general hospitals in Sharkia Province, where separate isolation units were devoted to COVID-19 patients. Sharkia Province, located in the Eastern Nile Delta, is considered the 2nd most populated province in Egypt, after the Great Cairo region, with about 8.0 million inhabitants.

### Study design and sampling

This was a cross-sectional study. The sample size was 360 physicians, as calculated according to the Epi Info 6.0, at 80% power of the study, 95% confidence level [12], as the total number of physicians who are currently working in governmental general hospitals in Sharkia province is 7759 [13]. We used a systematic random method, by selecting items from an ordered population utilizing a skip or sampling interval. During the data collection, 40 physicians (11.1%) refused to complete the study. After excluding the incomplete responses, the final sample size included in the study was 320 physicians. The survey was conducted from May 10th to June 9th, 2020. The data were collected via telephone interview due to lockdown measures, as the local health authorities banned face-to-face contact.

### Data collection and assessment tools

#### Exposure ascertainment

The main exposure variable was the perceived fear of COVID-19 virus infection (FCV) among physicians. It was assessed by using the Fear of COVID-19 Scale (FCV-19S) [14]. The FCV-19S is a new short valid seven-item psychometric scale assessing an individual’s fear of COVID-19. The participants indicate their level of agreement with the statements using a five-item Likert-type scale. Answers included “strongly disagree,” “disagree,” “neither agree nor disagree,” “agree,” and “strongly agree”. The minimum score possible for each question is 1 (strongly disagree), and the maximum is 5 (strongly agree). A total score is calculated by adding up each item score (ranging from 7 to 35). The higher the score, the greater the FCV. The Arabic version of this scale, used in this study, was translated, and its reliability and validity were examined [15].

#### Outcome ascertainment

The primary outcomes were the occupational symptoms of burnout syndrome (BOS) and the quality of life (QoL) of physicians. Symptoms of BOS were evaluated by the Maslach Burnout Inventory-Human Services Survey for Medical Personnel (MBI-HSS (MP) [6]. This inventory is a 22-item survey that covers 3 areas: emotional exhaustion (EE) (9 items), depersonalization (DP) (5 items), and personal accomplishment (PA) (8 items). Each subscale measures its unique dimension of BOS and includes multiple questions with frequency rating choices of never, a few times a year or less, once a month or less, a few times a month, once a week, a few times a week, or every day with scores from 0 to 6. The subscale total scores were 54 for EE, 48 for PA, and 30 for DP. The level of burnout was high if EE was ≥ 27, PA was ≤ 21, and DP was ≥ 13; moderate if EE was 17–26, PA was 38–22, and DP was 7–12; and low if EE was ≤ 16, PA was ≥ 39, and DP was ≤ 6. QoL of physicians was evaluated by the World Health Organization Quality of Life Scale (WHOQOL-BREF) [16]. This scale has been developed to provide a short-form QoL assessment. All participants were requested to answer the questions related to their lives during the past month, the period that was mostly encompassed during the outbreak. The scale contains a total of 26 questions based on a four-domain structure, which are physical health, psychological, social relationships, and environment. Two separately scored items assess overall QoL and General Health Satisfaction. Items are answered on a 5-point scale with a 2-week timeframe. The mean of items within each domain is multiplied by four to yield the domain score (range 4–20). Higher scores indicate higher QoL.

### Covariates

Demographic and clinical variables and associated depression and anxiety were considered as potential confounders in this study. A simple structured questionnaire was designed to collect relevant demographic (age, gender, marital status, residence, professional title, and occupation) and clinical data. Associated depression and anxiety were assessed using the Hospital Anxiety and Depression scale (HADS) [17]. HADS is a self-report rating scale of 14 items on a 4-point Likert scale (range 0–3). It is designed to measure anxiety and depression (7 items for each subscale). The total score is the sum of the 14 items, and for each subscale, the score is the sum of the respective seven items (ranging from 0–21). The Arabic version of this scale, used in this study, was translated and examined for its reliability and validity [18].

### Analysis methods

The SPSS (Statistical Package for Social Science) statistical software version 16.0 was used for data analysis. Quantitative data were represented as arithmetic means and standard deviations; the analysis of variance test (ANOVA) was used for comparison between two groups. Qualitative data were represented as frequencies and percentages; the Chi-square test (*χ*^2^) was carried out for calculating significant relations between the groups. To evaluate the degree of relationship between two variables with a linear relationship, the Pearson correlation coefficient was used. A nonparametric test (Mann-Whitney) was used to compare the means when data were not normally distributed. All results were considered statistically significant when the significant probability was less than 5% (*p* < 0.05), highly significant when the probability of error is less than 1% (*p* < 0.01), and very highly significant when the probability of error is less than 0.1% (*p* < 0.001).

## Results

The study included a total of 320 Egyptian physicians who were currently working and facing the outbreak of the COVID-19 pandemic in general hospitals in Sharkia Province, with a mean age of 34.6 ± 6.04 years. Most of them were females (*n* = 203, 63.4%) and married (*n* = 233, 72.8%). Frontline physicians, directly dealing with isolated patients diagnosed with COVID-19 virus infection, represented 15% of physicians (*n* = 48). Of note, receiving insufficient training related to pandemic and dissatisfaction with the hospital personal protective equipment (PPE) measures were reported by 72% (*n* = 229) and 83% (*n* = 266) of physicians, respectively. Regarding the psychiatric symptoms, ideas about death, moderate-to-severe anxiety, and depressive symptoms were reported by 11, 28, and 29% of physicians, respectively. For BOS symptoms, high emotional exhaustion, high depersonalization, and low personal accomplishment were reported by 20, 71, and 39% of physicians, respectively.

There were statistical differences between the mean scores of all items of BOS and age, anxiety and depression, ideas about death, and insufficient training related to pandemic among physicians, as shown in Table 1. Likewise, there were statistical differences between the mean scores of the four domains of QoL of physicians and anxiety and depression, insufficient training related to the pandemic, physicians’ dissatisfaction with their hospital PPE measures, history of medical colleague affection with COVID-19 infection, and history of mental illness, as shown in Table 2. Regarding the physicians’ FCV, the mean score of the fear of COVID-19 scale was positively correlated with those of anxiety, depression, and BOS emotional exhaustion and depersonalization subscales, and negatively correlated with those of personal accomplishment subscale and all QoL domains, as illustrated in Table 3. According to the physicians’ role, there were statistical differences between frontline and second-line physicians in terms of age, total depersonalization score, history of medical colleague affection with COVID-19 infection, and smoking, as shown in Table 4.

**Table 1:** Association between demographic and clinical characteristics of physicians and the three domains of Maslach Burnout Inventory

Maslach Burnout Inventory	Emotional exhaustion		Depersonalization	Personal accomplishment		
	Pearson correlation (***r***)					
**Age**	− 0.2**		− 0.3***	0.2**		
**Number of daily working hours**	0.2***		0.2***	0.2		
**Number of weekly working days**	0.1		0.1	0.1*		
**HADS**						
Total anxiety scores	0.5***		0.4***	-0.2**		
Total depression scores	0.4***		0.3***	-0.2**		
	**M (SD), MWU test**					
**Gender**						
Male	25.3 (9.6)	− 1.2	20.6 (9.3)	− 2.7**	28.8 (6.4)	− 2.9**
Female	24.0 (9.3)		17.9 (9.2)		27.4 (6.9)	
**Marital status**						
Non-married	25.4 (9.0)	− 1.1	20.5 (9.4)	− 1.9	27.7 (7.4)	− 0.4
Married	24.2 (9.5)		18.3 (9.2)		27.2 (6.7)	
**Title**						
Fresh graduate/resident	27.5 (8.3)	− 4.1***	22.5 (9.4)	− 4.7***	26.7 (6.9)	− 1.3
Specialist/consultant	23.0 (9.6)		17.1 (8.8)		27.7 (6.9)	
**Received training-related to pandemic**						
No	25.6 (9.1)	− 3.5***	19.9 (9.3)	− 2.9**	26.7 (6.6)	− 2.2*
Yes	21.6 (9.5)		16.6 (9.0)		28.8 (7.3)	
**Satisfied with the hospital infection control precautions**						
No	25.9 (9.2)	− 5.9***	19.9 (9.3)	− 4.2***	27.1 (6.9)	− 1.3
Yes	17.7 (7.5)		14.1 (7.5)		28.4 (6.8)	
**Physician’s role**						
2nd line physician	24.2 (9.5)	− 1.3	18.4 (9.2)	− 2.4*	27.4 (6.8)	-0.6
Frontline physician	26.2 (8.8)		22.0 (9.4)		26.8 (7.3)	
**History of medical colleague affection with COVID-19 virus infection**						
No	22.8 (8.9)	− 3.7***	17.1 (8.7)	− 3.8***	27.6 (7.0)	-0.8
Yes	26.7 (9.6)		21.3 (9.6)		27.0 (6.7)	
**History of mental illness**						
No	23.2 (9.3)	− 4.1***	18.1 (9.0)	− 2.5*	27.4 (6.9)	− 0.1
Yes	28.1 (8.8)		21.2 (9.7)		27.3 (7.0)	
**History of chronic medical illness**						
No	24.2 (9.3)	− 0.8	19.1 (9.3)	− 0.9	27.2 (7.0)	− 0.7
Yes	25.5 (9.9)		18.2 (9.2)		27.9 (6.5)	
**Smoking**						
No	24.2 (9.5)	− 1.7	18.5 (9.2)	− 2.6**	27.2 (6.9)	− 1.0
Yes	27.0 (8.3)		22.9 (9.1)		28.4 (6.7)	
**Ideas about death during pandemic**						
No	23.9 (9.3)	− 3.1**	18.3 (9.2)	− 3.5***	27.6 (7.0)	− 2.4*
Yes	29.2 (8.8)		24.2 (8.8)		24.9 (5.4)	

*HADS* Hospital Anxiety and Depression Scale

*p < 0.05, **p < 0.01, ***p < 0.001

**Table 2:** Association between demographic and clinical characteristics of physicians and the four domains of WHOQOL-BREF

WHOQOL-BREF	Physical health		Psychological health		Social relationship		Environmental	
	Pearson correlation (***r***)							
**Age**	0.06		0.11		− 0.01		0.05	
**Number of daily working hours**	− 0.05		− 0.09		− 0.01		− 0.17**	
**Number of weekly working days**	0.04		0.05		0.02		− 0.01	
**HADS**								
Total anxiety scores	− 0.65***		− 0.63***		− 0.49***		− 0.60***	
Total depression scores	− 0.62***		− 0.64***		− 0.49***		− 0.50***	
**M (SD), MWU test**								
**Gender**								
Male	55.8 (16.9)	− 4.4***	54.0 (19.0)	− 2.2*	56.4 (19.1)	− 1.0	48.6 (15.6)	− 0.8
Female	46.9 (16.1)		48.4 (18.9)		54.2 (18.9)		46.68 (16.2)	
**Marital status**								
Non-married	49.7 (16.5)	− 0.2	50.1 (20.1)	− 0.1	52.3 (18.3)	− 1.6	46.7 (16.9)	− 0.5
Married	50.3 (17.1)		50.6 (18.8)		55.9 (19.1)		47.7 (15.7)	
**Title**								
Fresh graduate/resident	48.0 (16.1)	− 1.8	45.6 (17.2)	− 3.1**	54.2 (18.9)	− 0.3	44.4 (15.3)	− 2.6*
Specialist/consultant	51.2 (17.2)		52.8 (19.6)		55.3 (19.0)		48.9 (16.2)	
**Received training-related to pandemic**								
No	48.3 (16.9)	− 2.8**	47.9 (18.4)	− 3.6***	52.6 (18.7)	− 3.6***	45.5 (15.8)	− 3.3**
Yes	54.6 (16.2)		56.8 (19.4)		60.8 (18.5)		52.2 (15.5)	
**Satisfied with the hospital infection control precautions**								
No	47.9 (16.2)	− 4.7***	48.2 (18.8)	− 4.7***	53.0 (18.7)	− 3.8***	44.8 (15.1)	− 6.2***
Yes	60.9 (16.3)		61.5 (17.0)		64.5 (17.6)		60.0 (14.5)	
**Physician’s role**								
2nd line physician	50.4 (16.6)	− 0.5	50.3 (19.1)	− 0.3	55.6 (18.8)	− 1.6	48.1 (15.9)	− 1.9
Frontline physician	48.4 (18.7)		51.4 (19.2)		51.54 (19.6)		43.5 (16.3)	
**History of medical colleague affection with COVID-19 virus infection**								
No	52.1 (16.0)	− 2.3*	51.52 (18.6)	− 1.3	56.6 (18.4)	− 2.0*	50.5 (15.2)	− 3.9***
Yes	47.5 (17.83)		49.0 (19.8)		52.7 (19.6)		43.2 (16.2)	
**History of mental illness**								
No	52.8 (15.9)	− 4.5***	52.8 (18.2)	− 3.9***	57.5 (17.9)	− 3.9***	49.50 (15.4)	− 4.2***
Yes	42.6 (17.5)		43.9 (20.1)		47.9 (20.2)		41.6 (16.4)	
**History of chronic medical illness**								
No	51.7 (16.0)	− 2.8**	50.9 (18.9)	− 0.8	56.4 (18.5)	− 2.5*	48.2 (15.9)	− 1.3
Yes	44.6 (19.0)		48.7 (19.8)		50.1 (19.8)		44.7 (16.3)	
**Smoking**								
No	49.6 (17.0)	− 1.8	50.5 (19.3)	− 0.4	55.1 (18.8)	− 0.1	47.5 (16.2)	− 0.4
Yes	54.9 (15.4)		49.6 (18.0)		54.2 (20.5)		46.4 (14.3)	
**Ideas about death during pandemic**								
No	51.2 (16.7)	− 3.4**	51.2 (19.0)	− 2.3*	55.6 (18.2)	− 1.5	48.3 (16.1)	− 3.5**
Yes	41.0 (16.1)		44.12 (19.4)		49.6 (24.0)		39.4 (12.6)	

*HADS* Hospital Anxiety and Depression Scale

*p < 0.05, **p < 0.01, ***p < 0.001

**Table 3:** Scores of anxiety, depression, burnout, and quality of life, and their correlation with the physicians’ fear from COVID-19 virus infection

FCV-19S score	M (SD)	Pearson correlation (***r***)	***P***
**HADS**			
Total anxiety scores	8.0 (4.5)	0.61	< 0.001
Total depression scores	9.1 (3.2)	0.50	< 0.001
**Maslach burnout inventory**			
Total emotional exhaustion	24.5 (9.4)	0.32	< 0.001
Total depersonalization	18.9 (9.3)	0.19	< 0.001
Total personal accomplishment	27.4 (6.9)	− 0.17	0.002
**WHOQOL-BREF**			
Physical health domain	50.1 (16.9)	− 0.45	< 0.001
Psychological health domain	50.4 (19.1)	− 0.38	< 0.001
Social relationship domain	55.0 (19.0)	− 0.26	< 0.001
Environmental domain	47.4 (16.0)	− 0.39	< 0.001

*FCV-19S* Fear of Covid-19 Scale, *HADS* Hospital Anxiety and Depression Scale

**Table 4:** Demographic and clinical characteristics according to the current physicians’ role

	Frontline physicians (***n*** = 48)	Second-line physicians (***n*** = 272)	***X***^***2***^ or ANOVA, ***P***
	M (SD)		
**Age**	32.3 (4.5)	35.0 (6.2)	8.5, 0.004
**Number of daily working hours**	9.2 (4.4)	8.2 (4.9)	1.8, 0.183
**Number of working days/w**	4.8 (1.5)	4.4 (1.5)	1.7, 0.196
**HADS**			
Total anxiety scores	9.1 (5.2)	7.8 (4.3)	3.5, 0.061
Total depression scores	9.2 (2.9)	9.0 (3.3)	0.6, 0.759
**FCV-19S score**	19.7 (3.3)	19.8 (5.1)	0.2, 0.995
**Maslach Burnout Inventory**			
Total emotional exhaustion score	26.2 (8.8)	24.2 (9.5)	1.8, 0.183
Total depersonalization score	22.0 (9.4)	18.4 (9.2)	6.3, 0.013
Total personal accomplishment score	26.8 (7.3)	27.4 (6.8)	0.3, 0.561
**WHOQOL-BREF**			
Physical Health domain	48.4 (18.7)	50.4 (16.6)	0.6, 0.451
Psychological health domain	51.4 (19.2)	50.3 (19.1)	0.1, 0.719
Social relationship domain	51.5 (19.6)	55.6 (18.8)	1.8, 0.176
Environmental domain	43.5 (16.3)	48.1 (15.9)	3.4, 0.065
	***N*** **(%)**		
**Gender**			
Male	21 (43.8)	96 (35.3)	1.3, 0.262
Female	27 (56.2)	176 (64.7)	
**Marital status**			
Non-married	18 (37.5)	69 (25.4)	3.0, 0.082
Married	30 (62.5)	203 (74.6)	
**Title**			
Fresh graduate/resident	21 (43.8)	85 (31.2)	2.9, 0.090
Specialist/consultant	27 (56.2)	187 (68.8)	
**Received training-related to pandemic**			
No	30 (62.5)	199 (73.2)	2.3, 0.131
Yes	18 (37.5)	73 (26.8)	
**Satisfied with the hospital infection control precautions**			
No	42 (87.5)	224 (82.4)	0.8, 0.380
Yes	6 (12.5)	48 (17.6)	
**History of medical colleague affection with COVID-19 virus infection**			
No	11 (22.9)	173 (63.6)	27.6, < 0.001
Yes	37 (77.1)	99 (36.4)	
**Smoking**			
No	35 (72.9)	251 (92.3)	16.1, < 0.001
Yes	13 (27.1)	21 (7.7)	
**Ideas about death during pandemic**			
No	40 (83.3)	246 (90.4)	2.2, 0.141
Yes	8 (16.7)	26 (9.6)	

*HADS* Hospital Anxiety and Depression Scale, *FCV-19S* Fear of Covid-19 Scale

## Discussion

In March 2020, the World Health Organization (WHO) announced COVID-19 outbreak as a global pandemic. This pandemic was associated with the emergence of new cases of depression and anxiety and/or an exacerbation of existing mental disorders, with distinguished psychological and physical impacts on health care providers [19]. The perceived extensive fear of COVID-19 (FCV) is a novel term that describes the anxiety and apprehensive thoughts of getting COVID-19 infection. This FCV would likely be attributed to the uncertainties about potential sequelae of the current outbreak [20].

The main finding of this study is that there was a robust correlation between the FCV and BOS symptoms (higher depersonalization and emotional exhaustion, and lower personal accomplishment) and poor QoL (physical, psychological, social, and environmental domains). Physicians often had conflictual thoughts and feelings about making a balance between their roles as healthcare providers with professional responsibility and the anxiety of being infected, with self-reproach about potentially infecting their families [21, 22]. A study investigating the possible etiologies of excessive fears among medical staff found that the FCV was attributed to the fears of COVID-19 complications reported by their patients (60.36%), infecting their family members (80%), misdiagnosing their COVID-19 patients (28%), or becoming asymptomatic carriers (29%). These disturbed perceptions regarding the pandemic were a stressful state that would eventually increase the risk of developing BOS symptoms [23]. Moreover, several studies reported that FCV had a negative impact on the psychological and physical well-being of the individuals particularly during the periods of lockdown by intensifying the levels of depression, anxiety, and perceived stress, and reducing sleep quality [24, 25]. During the current crisis, thousands of healthcare providers including physicians were infected or even lost their lives worldwide [26], with an immense toll on the health care system during the current pandemic, and most physicians experienced higher levels of extreme workloads, associated with a state of uncertainty, health anxiety, and emotional distress as most physicians had insufficient time to take care of their families during the epidemic [23].

In Egypt, during the time of data collection, the official report of the Egyptian Ministry of Health and Population referred that the confirmed COVID-19 infected cases reached about 48,000 cases and around 1800 deaths (mortality rate 2–3%). The numbers of physicians and other health care workers who were infected and deceased, however, were extremely high when compared to the general population. The official number of infected doctors was about 430 with 68 deaths (mortality rate 16%) [27]. A recent study found that the most frequent concerns regarding the COVID-19-related fears among medical staff in Egypt were the high transmissibility of the disease, the fears of getting infected and transmitting the infection to their families, and the COVID-19-related stigma. Furthermore, the workplace-related factors would add further loads to the physicians. It was claimed that the limited numbers of well-trained physicians and other health care workers, insufficient personal protective equipment (PPE), dealing with the public who were not committed to protective or social distance measures together with ill-ventilated and overcrowded workplaces were potential causes of increased levels of anxiety among Egyptian physicians [28].

Previous research reported that BOS might be complicated by depression, social isolation, and suicide among physicians [29, 30]. The current study found that associated anxiety and depression, ideas about death, and insufficient COVID-19 training were associated with all items of BOS among physicians. It was unclear whether such variables were etiologies or consequences of BOS. Yet, it was likely that both scenarios might occur [31, 32]. Based on the research of previous outbreaks of Middle East respiratory syndrome (MERS), severe acute respiratory syndrome (SARS), influenza, and H1N1, it was well established that young physicians experienced a varying degree of BOS. Anxiety and stress developed during the outbreaks were found to be associated with higher Maslach burnout inventory scores [33, 34]. Also, the lack of effective training and inadequate personal protective equipment (PPE) were reported as major contributing factors [33].

Interestingly, this study stated that being fresh graduates and residents was associated with higher scores of emotional exhaustion and depersonalization subscales of BOS. Our findings were in line with previous studies conducted in nearby Arabic [35] and non-Arabic countries during previous pandemics [36, 37]. The younger physicians would be more vulnerable to BOS owing to the heavier workloads, unpredicted changes in duty schedules to accommodate the new needs, cancelation of vacations, and prolonged contact with COVID-19 patients [38].

Frontline physicians were the ones who had direct contact with isolated patients diagnosed with COVID-19 virus infection. The current study revealed that the frontline physicians were more likely to be younger and develop depersonalization symptoms than second-line physicians. To ensure gaining clinical skills and accommodate to work under a higher level of stress, residents and young physicians were expected to spend a substantial part of their residency programs at the emergency departments, which would increase the probability of contact with COVID-19 patients. Moreover, frontline physicians were more likely to use their PPE for a long time resulting in excessive heat and lack of hydration and alimentation which would affect their physical health status. Therefore, all together with sleep deprivation and accentuated fatigue would eventually lead to the development of BOS symptoms [39]. Unlikely, another Chinese study, however, found that the frontline health professionals reported a milder degree of BOS symptoms than their second-line counterparts during COVID-19 pandemic. This apparent difference was explained as frontline physicians might perceive a greater sense of control of their situation, which would act as the main driver of engagement and essential for overcoming BOS [40].

### Limitations

The study was a cross-sectional study; therefore, it was difficult to investigate the cause-effect relationship. Besides, the physicians’ subspecialty as a potential variable was not assessed. However, our study had several strengths; to our knowledge, this study would be one of the earliest studies, if any, in Egypt which investigated the impact of FCV during the peak of the pandemic. This would lead this study to serve as one of the few sources of information about the mental health impacts of the COVID-19 pandemic in Egypt.

## Conclusions

There was a positive correlation between FCV and associated anxiety, depression, higher levels of BOS, and poor QOL among physicians in Egypt. Poor QoL was linked to associated anxiety or depression, being an untrained physician to deal with COVID-19 or unsatisfied with hospital infection control measures, or having a history of mental illness. Plans, including proper multifaceted training programs and crisis management policies for such events, should be adopted to minimize the physical and mental burdens on the physicians.

## Data Availability

The datasets used and/or analyzed during the current study are available from the corresponding author on reasonable request.

## Abbreviations

FCV: Fear of COVID-19 virus infection
QoL: Quality of life
BOS: Burnout syndrome
HADS: Hospital Anxiety and Depression scale
FCV-19S: Fear of COVID-19 scale
WHOQOL-BREF: World Health Organization Quality of Life Scale
MBI-HSS (MP): Maslach Burnout Inventory–Human Services Survey for Medical Personnel
EE: Emotional Exhaustion
DP: Depersonalization
PA: Personal accomplishment
PPE: Personal protective equipment
